# Supplementary material for: Indications of the SERPINE 1 variant rs1799768’s role in anti-VEGF therapy resistance in neovascular age-related macular degeneration
Source: PLoS One. 2025 Mar 6;20(3):e0317511. doi: 10.1371/journal.pone.0317511 (PMC11884677; doi:10.1371/journal.pone.0317511)
Supplement: S1 Table — F indicates female patients, M indicates male patients. (DOCX) [file pone.0317511.s001.docx]

|  | AGE (YEARS) | | FOLLOW-UP (MONTHS) | | INJECTION TIMES | |
| --- | --- | --- | --- | --- | --- | --- |
| PATIENT | OPTIMAL | SUBOPTIMAL | OPTIMAL | SUBOPTIMAL | OPTIMAL | SUBOPTIMAL |
| F1 | 76.1 | 61.0 | 19.1 | 24.1 | 2 | 11 |
| F2 | 74.1 | 62.0 | 13.4 | 20.6 | 8 | 9 |
| F3 | 67.2 | 88.0 | 16.8 | 27 | 6 | 16 |
| F4 | 71.0 | 79.0 | 19.3 | 20 | 8 | 11 |
| F5 | 86.0 | 77.0 | 14.4 | 24.9 | 7 | 11 |
| M1 | 60.0 | 65.0 | 20.3 | 17.5 | 2 | 9 |
| M2 | 77.1 | 64.0 | 16.5 | 19.6 | 10 | 18 |
| M3 | 76.0 | 80.0 | 17.7 | 24.9 | 7 | 13 |
| M4 | 67.5 | 74.0 | 16.5 | 24.8 | 7 | 17 |
| M5 | 67.0 | 88.0 | 25.9 | 26.7 | 9 | 15 |
| AVG | 72.2 | 73.8 | 17.99 | 23.27777778 | 6.6 | 13 |
| STD | 7.24277418 | 10.3 | 3.50791169 | 3.381485538 | 2.67498702 | 3.299831646 |
