# Supplementary material for: Indications of the SERPINE 1 variant rs1799768’s role in anti-VEGF therapy resistance in neovascular age-related macular degeneration
Source: PLoS One. 2025 Mar 6;20(3):e0317511. doi: 10.1371/journal.pone.0317511 (PMC11884677; doi:10.1371/journal.pone.0317511)
Supplement: S2 Table — ‘Optimal’ refers to responsive patients, ‘suboptimal’ refers to suboptimal responsive patients. (DOCX) [file pone.0317511.s002.docx]

| POLYMORPHISMS  PATIENTS | FV LEIDEN (G1691A) | FII (G20210A) | MTHFR  (C677T) | MTHFR (A1298C) | PAI-1  4G/5G |
| --- | --- | --- | --- | --- | --- |
| OPTIMAL1 | N | N | N | HET | 5G/5G |
| OPTIMAL2 | N | N | N | N | 5G/5G |
| OPTIMAL3 | N | N | N | HET | 5G/5G |
| OPTIMAL4 | N | N | N | N | 5G/5G |
| OPTIMAL5 | N | N | N | N | 5G/5G |
| OPTIMAL6 | N | N | N | N | 5G/5G |
| OPTIMAL7 | N | N | N | N | 4G/5G |
| OPTIMAL8 | N | N | N | HET | 4G/5G |
| OPTIMAL9 | N | N | HET | N | 5G/5G |
| OPTIMAL10 | N | N | HET | N | 5G/5G |
| SUBOPTIMAL1 | N | N | N | HET | 4G/4G |
| SUBOPTIMAL2 | N | N | HET | N | 4G/4G |
| SUBOPTIMAL3 | N | N | N | HET | 4G/4G |
| SUBOPTIMAL4 | N | N | N | HET | 4G/4G |
| SUBOPTIMAL5 | N | N | HET | N | 4G/5G |
| SUBOPTIMAL6 | N | N | HET | N | 4G/5G |
| SUBOPTIMAL7 | N | N | HET | N | 4G/5G |
| SUBOPTIMAL8 | HET | N | N | HOM | 5G/5G |
| SUBOPTIMAL9 | HET | N | HOM | HET | 5G/5G |
| SUBOPTIMAL10 | N | N | HET | HET | 4G/5G |

**S2 Table. Genotyping results.** ‘Optimal’ refers to responsive patients, ‘suboptimal’ refers to suboptimal responsive patients.
